# Application of Herbal Traditional Chinese Medicine in the Treatment of Acute Kidney Injury

**DOI:** 10.3389/fphar.2019.00376

**Published:** 2019-04-18

**Authors:** Hai-Di Li, Xiao-Ming Meng, Cheng Huang, Lei Zhang, Xiong-Wen Lv, Jun Li

**Affiliations:** ^1^The Key Laboratory of Major Autoimmune Diseases, Anhui Institute of Innovative Drugs, School of Pharmacy, Anhui Medical University, Hefei, China; ^2^The Key Laboratory of Anti-inflammatory and Immune Medicine, Ministry of Education, Anhui Medical University, Hefei, China; ^3^Institute for Liver Diseases, Anhui Medical University, Hefei, China; ^4^Anhui Key Laboratory of Bioactivity of Natural Products, School of Pharmacy, Anhui Medical University, Hefei, China

**Keywords:** acute kidney injury (AKI), traditional Chinese medicine (TCM), inflammation, apoptosis, nephrotoxicity

## Abstract

Acute kidney injury (AKI) is a clinical syndrome characterized by a rapid loss of renal function, which may further develop into chronic kidney damage (CKD) or even end-stage renal disease (ESRD). AKI is a global health problem associated with high morbidity and costly treatments, and there is no specific or effective strategy to treat AKI. In recent years, Traditional Chinese Medicine (TCM) has attracted more attention, with lines of evidence showing that application of TCM improved AKI, and the mechanisms of action for some TCMs have been well illustrated. However, reviews summarizing the progress in this field are still lacking. In this paper, we reviewed TCM preparations and TCM monomers in the treatment of AKI over the last 10 years, describing their renal protective effects and mechanisms of action, including alleviating inflammation, programmed cell death, necrosis, and reactive oxygen species. By focusing on the mechanisms of TCMs to improve renal function, we provide effective complementary evidence to promote the development of TCMs to treat AKI. Moreover, we also summarized TCMs with nephrotoxicity, which provides a more comprehensive understanding of TCMs in the treatment of AKI. This review may provide a theoretical basis for the clinical application of TCMs in the future.

## Introduction

Acute kidney injury (AKI), characterized by an abrupt decline of renal function, can be induced by numerous causes including renal ischemia reperfusion injury (IRI), nephrotoxic insults and infection of sepsis (Waikar et al., 2008; Linkermann, 2016). Accumulating evidence shows that AKI is a global public health concern and a pivotal threat to human health, especially in hospitalized patients, as it impacts more than 13 million patients per year (Chertow et al., 2005; Lameire et al., 2013; Thomas et al., 2015; Yang et al., 2015; Allison, 2016). Excessive inflammatory responses, oxidative stress, and the imbalance of the damage and repair of renal tubules, are all highly involved in the pathological process of AKI, however, specific targets and effective therapies are still lacking (Sancho-Martinez et al., 2015; Yang et al., 2016; Zuk and Bonventre, 2016).

Traditional Chinese medicine (TCM) has been widely used for the treatment of AKI and its complications in China and neighboring countries, including Japan and Korea, for a long time. Some TCM-based therapies show good results and high efficacy in inhibiting inflammatory responses, programmed cell death and oxidative stress. In this regard, the therapeutic effects of TCMs have widely been tested in animal models of AKI and even in patients. For instance, the Xuebijing Injection is effective in improving clinical symptoms of sepsis-induced AKI patients after the Wenchuan Earthquake (Yuxi et al., 2017). Our recent study showed that wogonin not only protects cisplatin-induced AKI, but also preserves and even promotes the anti-tumor effect of cisplatin (Meng et al., 2018). However, it is noteworthy that some TCMs, such as aristolochic acids and other plant alkaloids, are nephrotoxic, (Yang et al., 2018). So, the application of TCM should be carefully evaluated.

In this paper, we reviewed the therapeutic effects of TCMs on AKI and the mechanism of action based on the assessment of evidence that supports hypotheses, additionally, TCMs with nephrotoxicity have also been discussed.

## Applications of TCM in AKI

### TCM Preparations in AKI

Until now, several TCM preparations have been tested in the treatment of AKI. There are shown in Table 1.

**Table 1:** Application of TCM Preparations in the treatment of acute kidney injury.

Names	Origins	Models	Function	Mechanisms
A&A	*Astragalus membranaceus* var. *mongholicus* and *Angelica sinensis*	I/R-induced kidney injury	Decreasing cell necrosis	By inducing JNK (Cai et al., 2001; Meng et al., 2007)
DFD	*Radix et Rhizoma Rhei*, *Radix Aconiti Lateralis Praeparata*, and *Radix et Rhizoma Asari*	Adenine-induced renal injury	Inhibiting apoptosis	By blocking TGF-β1-JNK (Tu et al., 2014)
Xuebijing injection	*Radix paeoniae rubra*, *Chuan dome*, *Salvia miltiorrhiza*, *Safflower*, and *Chinese angelica*	Serious scald-induced renal injury	Alleviating renal function	By suppressing HMGB1 (Wang et al., 2007)
HLJDD	*Rhizoma coptidis* (*RC*), *Cortex phellodendri* (*CP*), *Radix scutellariae* (*RS*), and *Fructus gardenia*	LPS-induced AKI	Attenuating apoptosis	Activating the Akt/HO-1 pathway and inhibiting NF-kB and MAPK activation (Li et al., 2017)
ZDW	*Cornus officinalis* Siebold & Zucc., *Radix Rehmanniae preparata*, *Dioscorea oppositifolia* L., *Cortex Phellodendri*, *rhizome*, *Moutan Cortex*, *Rhizoma Alismatis*, and *Wolf*	Gentamicin-induced renal injury	Attenuating apoptosis	By limiting caspase-3 activation (Hsu et al., 2014)


#### *Astragalus membranaceus* var. *mongholicus* and *Angelica sinensis* (A&A)

Decoctions of roots from A&A can improve renal blood flow in a murine model of acute ischemic renal injury, possibly by increasing NO production by activating eNOS and scavenging ROS, therefore accelerating renal repair after ischemic injury (Meng et al., 2007). Moreover, the therapeutic effect of A&A may be JNK-dependent (Cai et al., 2001).

#### Dahuang Fuzi Decoction (DFD)

Dahuang Fuzi Decoction (DFD) consists of *Radix et Rhizoma Rhei*, *Radix Aconiti Lateralis Praeparata*, and *Radix et Rhizoma Asari*. Emerging evidence indicates that DFD attenuates adenine-triggered renal damage and tubular epithelial apoptosis, by blocking the activation of TGF-β1-JNK pathways (Tu et al., 2014).

#### Xuebijing Injection

It consists of *chuan dome*, *radix paeoniae rubra*, *safflower*, *Salvia miltiorrhiza*, and *Chinese angelica*. Administration of a Xuebijing injection can suppress the production and release of high mobility group box-1 protein (HMGB1) in the kidney, thereby alleviating serious scald injury-induced AKI (Wang et al., 2007). In addition, an intravenous injection of Xuebijing attenuates the inflammatory response in AKI rats with paraquat poisoning (Xu et al., 2017). Importantly, Xuebijing improved the clinical symptoms of patients with sepsis-induced AKI after the Wenchuan Earthquake (Yuxi et al., 2017).

#### Huang-Lian-Jie-Du-Decoction (HLJDD)

It is composed of *Rhizoma coptidis* (*RC*), *Cortex phellodendri* (*CP*), *Radix scutellariae* (*RS*), and *Fructus gardenia* by a weight ratio of 3:2:2:3. HLJDD effectively suppresses LPS-induced AKI by activating Akt/HO-1 pathway and inhibiting NF-κB and MAPK activation in mice (Li et al., 2017).

#### Zhibai Dihuang Wan (ZDW)

ZDW is a polyherbal formula mixed with *Rehmannia glutinosa* (Gaertn.) *DC*, *baked* (*Radix Rehmanniae preparata*), *Cornus officinalis* Siebold & Zucc., *Dioscorea oppositifolia* L., *Paeonia suffruticosa* Andrews, *Alisma plantago-aquatica* L., *rhizome* (*Rhizoma Alismatis*), and *Poria cocos* (Schw.) Wolf. ZDW has been used to treat chronic kidney diseases, like diabetic nephropathy, for many years. A recent study revealed that ZDW also protected against gentamicin-induced AKI both *in vivo* and *in vitro*, because it attenuated apoptosis of renal tubular epithelial cells by limiting caspase-3 activation (Hsu et al., 2014).

### TCM Monomers in AKI

Compared with TCM preparations, TCM Monomers have recently attracted more attention in the treatment of diseases because they have certain molecular structures, clear mechanisms of action, predicted pharmacological effects and less drug-drug interactions. In the kidney, numerous TCM monomers have been applied in treating renal diseases including AKI caused by different stimuli. Therefore, we list TCMs that have comprehensively been studied to protect against AKI in recent years.

#### Astaxanthin (ATX)

Astaxanthin (ATX) is a natural carotenoid extracted from marine organisms which are widely applied because of their strong antioxidant effect. Current studies demonstrate the renoprotective effects of ATX in many AKI models. ATX (5 mg/kg for 14 days via oral gavage) can improve I/R-induced AKI by exerting antioxidant activity and inhibiting tubular apoptosis/necrosis via scavenging free radicals (Qiu et al., 2015). Moreover, ATX (40 mg/kg for 5 days by intraperitoneal injection) attenuated arsenic-induced AKI by fulfilling antioxidant functions and reducing As accumulation (Wang et al., 2014), and ATX (50 mg/kg for 12 h by gavage) ameliorated HgCl_2_-induecd AKI by exerting anti-oxidant activity and preventing lipid and protein oxidation (Augusti et al., 2008). ATX (20 mg/kg 12 h via tail intravenous injection) consistently improved early AKI, following a severe burn, by modulating antioxidant activity and Akt/Bad/Caspases-mediated mitochondrial-apoptotic pathway (Guo et al., 2015).

#### Baicalin

Baicalin is a *Scutellaria baicalensis*-derived flavonoid which has been tested in multiple types of AKI models. In clinical trials, Baicalin protects against AKI in pediatric sepsis by inhibiting renal cell apoptosis (Zhu et al., 2016). In ischemia-reperfusion injured kidney, Baicalin (10 μmol/L for 24 h) exerts protective effects by inhibiting TLR2/4-mediated inflammation and mitochondrial stress-induced apoptosis of tubular epithelial cells (Ji et al., 2014). Administration of Baicalin (100 μmol/L) in HK-2 cells consistently reduced H_2_O_2_-induced cytotoxicity by activating downstream Nrf2 signaling and attenuating ER stress (Lin et al., 2014). These findings are supported by recent findings that Baicalin (50 mg/kg, i.p. for 2 weeks) prevents Lead (Pb)-induced renal injury and pediatric sepsis-induced AKI by blocking oxidative stress and apoptosis (Zhang et al., 2016; Zhu et al., 2016). Moreover, it is of note that baicalin is a novel PPAR-γ activator which may suppress NF-κB-mediated inflammation effectively (Lim et al., 2012).

#### *Cordyceps sinensis* (CS)

*Cordyceps sinensis* (CS) is used as a tonic food which is derived from an entomogenous fungus (Zhu et al., 1998). CS (5 g/kg via intragastric for 2 days) improves the outcome of I/R-induced AKI via different mechanisms including modulating SDF-1/CXCR4-signaling axis (Wang et al., 2013), up-regulating expression of HIF-1α, down-regulating the expression of NGAL (Yu et al., 2012) and reducing the expression level of TLR-4 (Zhou and Hu, 2010). Moreover, treatment of CS (1.5 mg/200 μl) significantly alleviates stress responses and tissue damage by reducing autophagy and apoptosis in LPS-induced AKI (Wu et al., 2011). Additionally, CSP, as the mycelia glycoproteins of *Cordyceps sobolifera*, significantly suppresses cyclosporine A (CsA)-induced apoptosis and protects against nephron loss via increasing magnesium reabsorption (Chyau et al., 2014).

#### Epigallocatechin Gallate (EGCG)

As a major component of green tea, EGCG is famous for its anti-inflammatory and anti-apoptotic properties. EGCG, as a potent inducer of HO-1, can suppress renal injury by reducing oxidative stress and inflammation in several AKI models induced by contrast (EGCG 20 mg/kg intravenously) (Gao Z. et al., 2016), I/R (EGCG 50 mg/kg i.p. for 24 h) (Lv et al., 2015) and cisplatin (EGCG 100 mg/kg i.p. for 12 days) (Sahin et al., 2010), respectively. Furthermore, underlying mechanisms have extensively been explored in cisplatin nephropathy, EGCG (100 mg/kg i.p. for 2 days) prevented activation of ERK, the NF-κB pathway and caspase-12 while down-regulating the Fas-conducted extrinsic pathway and Bcl-2/Bax ratio, thereby reducing the apoptosis of tubular epithelial cells (Zou et al., 2014; Chen B. et al., 2015; Pan et al., 2015).

#### Ginsenoside Rd (GSRd)

Ginsenoside Rd (GSRd) is isolated from the root of *Panax ginseng* and applied to protect cells in various types of diseases especially ischemia diseases (Ye et al., 2011). It is noteworthy that GSRd has an impact on different cell types which are involved in AKI. For instance, previous studies identified that GSRd (50 mg/kg i.p. for 2 days) prevented M1 macrophage polarization in I/R-injured kidney (Ren et al., 2016). Additionally, GSRd (5 mg/kg i.p. for 30 days) protected proximal tubule cells against I/R model-induced hypoxia-reoxygenation by inhibiting oxygen free radicals from attacking cell membranes (Yokozawa et al., 1998). The renoprotective effect of GSRd (5 mg/kg i.p. for 30 days) was further determined in cisplatin and glycerol-induced AKI models, treatment of GSRd decreased apoptosis-triggered DNA fragmentation and oxidative stress (Yokozawa and Liu, 2000; Yokozawa and Dong, 2001; Zhou et al., 2014). Other Ginsenosides, such as Rb1, Rg1 (80 mg/L for 24 h) and Rg3 also proved to be effective in the treatment of AKI. It has been identified that administration of Ginsenoside Rb1 relieves apoptosis of HK-2 cells in response to serum from I/R AKI (Zhu et al., 2009). Ginsenoside Rg1 reduces aldosterone-induced oxidative stress and abnormal autophagy correlates with AMPK/mTOR pathway. Ginsenosides 20(S)-Rg3 exerts therapeutic effects in both cisplatin (GSRd 250 μg/mL for 24 h) and LPS (GSRd 10 mg/kg i.p. for 15 days)-induced AKI by targeting JNK-p53-caspase-3 axis and NF-κB signaling pathway (Kang et al., 2007; Wang et al., 2015; Han et al., 2016).

#### Resveratrol (RSV)

Resveratrol (RSV), a popular natural phenolic compound which is abundant in wines and grape skins, protects against multiple types of AKI due to its low toxicity, powerful antioxidants, and anti-inflammatory properties. Resveratrol (100 mg/kg for 20 h by oral gavage) can attenuate LPS-induced AKI by suppressing inflammation and apoptosis driven by macrophages (Chen L. et al., 2015). Resveratrol (10 mg/kg i.p. for 12 h) consistently protects against sepsis-induced tubular epithelium injury by restoring the renal microcirculation and scavenging reactive nitrogen species (Holthoff et al., 2012). In addition, resveratrol (3 mg/kg for 6 days via the forearm vein) ameliorates arsenic trioxide (As_2_O_3_)-induced nephrotoxicity by antagonizing oxidative stress and facilitating arsenic metabolism (Yu et al., 2013). Moreover, resveratrol is proven to be an anti-inflammatory agent in glycerol (RSV 25 mg/kg/day for 4 days via gastric intubation)- and cisplatin (RSV 25 mg/kg/day i.p. for 2/5 days)-induced AKI (de Jesus Soares et al., 2007; Do Amaral et al., 2008). Furthermore, previous studies demonstrated that resveratrol-mediated activation of SIRT1 improved cisplatin (RSV 10 mg/kg orally once a day for 7 days)-induced AKI by deacetylating p53 and reducing apoptosis (Kim et al., 2011), and RSV also inhibited sepsis (RSV 10 mg/kg i.p. for 3 days)-induced AKI and renal inflammation through NF-κB de-acetylation (Gan et al., 2017) or SIRT3-mediated deacetylation of SOD2 (Xu et al., 2016). Resveratrol (30 mg/kg i.p. for 12 h) protected against early sepsis-induced AKI by inhibiting the endoplasmic reticulum stress (IRE1)-activated NF-κB pathway (Wang et al., 2017). A previous study showed that RSVA405 (3 mg/kg i.p. for 24 h) and RSVA314 (3 mg/kg i.p. for 24 h), two biologically active resveratrol analogs (RSVAs), attenuated I/R-induced AKI by exerting anti-oxidative and anti-inflammatory effects, indicating that RSV and its derivatives may be promising agents to prevent and/or treat AKI with high efficiency (Khader et al., 2015).

#### Tetramethylpyrazine (TMP)

Tetramethylpyrazine (TMP) is a natural product isolated from the Chinese herb *Ligusticum wallichii* Franch., which is famous for its antioxidative and anti-inflammatory effects. Previous studies showed that treatment with TMP protects against arsenic (TMP 100 μM for 6 h)-induced nephrotoxicity by targeting HO-1 and ARS2, which was further evidenced by the findings that TMP (20 mg/kg/day i.p. for 7 days) relieves gentamicin-induced AKI by enhancing Hax-1 mitochondrial localization in HO-1-dependent mechanisms (Sue et al., 2009; Gong et al., 2016). Additionally, by suppressing ROS production and the consequential inflammatory response, TMP protected against cisplatin (80 mg/kg/day orally for 7 days) or arsenic (100 μM for 24 h)-induced AKI (Ali et al., 2008; Gong et al., 2015). Moreover, a recent study showed that TMP (80 mg/kg/day i.p. for 4 days) suppressed the apoptosis of renal cells by targeting FoxO1, a pro-apoptotic transcription factor, to prevent contrast-induced AKI (Gong et al., 2013). Interestingly, TMP exerted a renoprotective role by downregulating P-selectin, which has been accepted as a key modulator of neutrophil infiltration in I/R kidney injury (Chen et al., 2003).

Our group also tested the therapeutic potential of traditional Chinese medicine in the treatment of AKI. We screened 10 kinds of Chinese herbal medicine with anti-inflammatory effects and found that wogonin and protocatechuic aldehyde had significant therapeutic effects. Wogonin inhibits cisplatin-induced renal damage by inhibiting RIPK1-mediated necroptosis and attenuates inflammation (Meng et al., 2018), whereas protocatechuic aldehyde (PA) not only inhibits necroptosis, but also effectively reduces cisplatin-induced over-production of ROS (Gao L. et al., 2016). Interestingly, we all know that cisplatin is commonly used as an anti-cancer drugs in clinic, and these two TCMs could even promote anti-tumor effects of cisplatin, so wogonin and protocatechuic aldehyde may be renoprotective adjuvants for cisplatin-based anticancer therapy.

There are many other TCMs to treat AKI, and these are listed in Table 2.

**Table 2:** Application of TCM monomers in the treatment of acute kidney injury.

Names	Origins	Models	Functions	Mechanisms
Alpinetin	*Alpinia katsumadai* Hayata	LPS-induced AKI	Inhibiting inflammation.	By enhancing Nrf2 and HO-1 (Huang et al., 2015)
Astragaloside IV (AS-IV)	*Astragaloside*	Cisplatin-induced AKI	Inhibiting oxidative damage and inflammatory response.	By activation of Nrf2 and suppression of NF-κB activation (Yan et al., 2017)
Astaxanthin (ATX)	*Carotenoid in marine organisms*	I/R, As_2_O_3_, HgCl_2_-induced AKI	Antioxidant activity; Inhibiting apoptosis.	By Akt/Bad/caspases pathway (Augusti et al., 2008; Wang et al., 2014; Guo et al., 2015; Qiu et al., 2015)
Baicalin	*Scutellaria baicalensis*	H_2_O_2_, -induced AKI	Blocking oxidative stress, ER stress and apoptosis.	By activating Nrf2 signaling (Lin et al., 2014)
		Pb, pediatric sepsis – induced AKI		Zhang Z. et al., 2017
		I/R-induced AKI	Inhibiting inflammation and apoptosis.	By inhibiting TLR2/4 and mitochondrial stress (Ji et al., 2014)
		LPS-induced AKI		By activating PPARγ and inhibiting NF-κB (Lim et al., 2012)
Breviscapine	*Erigeron breviscapus*	Cisplatin-induced AKI	Inhibiting lipid peroxidation and ferroptosis.	By decreasing MDA, SOD, increasing glutathione peroxidase levels (Lou et al., 2015)
Chlorogenic Acid	*Plant polyphenols*	LPS-induced AKI	Suppressing inflammation.	By inhibiting TLR4/NF-κB signaling pathway (Ye et al., 2017)
*Cordyceps sinensis* (CS)	*An entomogenous fungus*	I/R-induced renal injury	Inhibiting inflammation and apoptosis.	By modulating SDF-1/CXCR4-signaling, reducing TLR-4,increasing HIF-1α (Zhou and Hu, 2010; Yu et al., 2012; Wang et al., 2013)
		LPS-induced AKI	Reducing autophagy and apoptosis.	By reducing ED-1, GRP78 (Wu et al., 2011)
(CSP)	*Cordyceps sobolifera*	CsA – induced AKI	Suppressing apoptosis.	By enhancing TRMP6 and TRMP7 (Chyau et al., 2014)
Curcumin	*Curcuma longa*	Rhabdomyolysis (RM)-induced AKI	Reducing renal oxidative stress.	By inhibiting AMPK and Nrf2/HO-1 (Wu et al., 2017)
		I/R-induced AKI		By NMDA receptor antagonism (Kaur et al., 2016)
		Glycerol-induced AKI	Ameliorating cell apoptosis.	By activating the PI3K/Akt pathway (Wu et al., 2017)
		Cisplatin-induced AKI	Preventing renal alterations. Inhibiting inflammatory.	By preventing mitochondrial bioenergetics and dynamic and SIRT3 levels (Ortega-Dominguez et al., 2017). By inhibiting Mincle-maintained M1 macrophage phenotype (Tan et al., 2019)
Emodin	*Rheum palmatum*	LPS-induced AKI	Inhibiting inflammatory.	By inhibiting TLR2 (Li et al., 2015) or TLR4 (Zhu et al., 2012)
		Cisplatin-induced AKI	Inhibiting apoptosis and activating autophagy.	By modulating the AMPK/mTOR signaling (Liu et al., 2016)
Epigallocatechin gallate (EGCG)	*Green tea*	Contrast-induced AKI	Alleviating apoptosis, oxidative stress and inflammation.	By increasing HO-1 and Nrf2 (Gao Z. et al., 2016)
		I/R, Cisplatin -induced AKI	Inhibiting inflammatory, Decreasing oxidative/nitrative stress.	By activating HO-1 (Sahin et al., 2010; Lv et al., 2015; Pan et al., 2015)
			Inhibiting apoptosis.	By preventing ERK (Zou et al., 2014)
Ginsenoside Rd (GSRd)	*Panax ginseng*	I/R-induced AKI	Suppressing inflammatory.	By inhibiting oxygen free radicals (Ye et al., 2011)
		Cisplatin-induced AKI	Decreasing apoptosis.	Yokozawa and Liu, 2000
		Glycerol-induced AKI	Reducing renal oxidative stress.	Zhou et al., 2014
(Rb1, Rg1)		I/R-induced AKI	Reducing apoptosis.	Zhu et al., 2009
(Rg1)		Aldosterone- induced AKI	Reducing oxidative stress and autophagy.	By decreasing AMPK/mTOR pathway (Wang et al., 2015)
(Ginsenoside Rg3)	*Panax ginseng*	Cisplatin-induced AKI	Decreasing apoptosis.	By blocking the JNK-p53-caspase-3 signaling (Han et al., 2016)
		LPS-induced AKI	Decreasing inflammatory.	By inhibiting NF-κB (Kang et al., 2007)
Esculentoside A (EsA)	*Phytolacca esculenta*	LPS-induced AKI	Alleviating inflammation.	By activating PPAR-γ (Chen et al., 2017)
		Puncture-induced AKI		By regulating the TLR4/MyD88/HMGB1 signaling pathway (Sun et al., 2017)
Galangin	*Propolis and Alpinia officinarum*	Cisplatin-induced AKI	Attenuating oxidative stress, inflammation, and cell death.	By inhibiting ERK, NF-κB and RIPK1-mediated necroptosis signaling pathways (Huang et al., 2017)
Ginkgetin aglycone (GA)	*Ginkgo biloba extract*	LPS-induced AKI	Decreasing inflammatory.	By activating SIRT1 via inhibiting the NF-κB signaling pathway (Zhang J. et al., 2017)
Glycyrrhizic acid (GA)	*Ingredient in licorice*	LPS-induced renal injury	Inhibiting cell apoptosis, oxidative stress.	By activating ERK and inhibiting NF-κB (Zhao et al., 2016)
		I/R-induced renal injury	Reducing tubular necrosis.	By inhibiting HMGB1 and enhancing Nrf2 (Lau et al., 2014)
(GA, 18βGA)		Cisplatin-induced AKI	Inhibiting renal tubular epithelial cells apoptosis.	By enhancing BMP-7 epigenetically through targeting HDAC2 (Ma et al., 2016)
			Alleviating oxidative status and inflammatory.	Arjumand and Sultana, 2011; Wu et al., 2015
Gypenoside (GP)	*Gynostemma pentaphyllum*	I/R-induced renal injury	Attenuating inflammatory and oxidative stress.	By inhibiting ERK signaling (Ye et al., 2016)
Hyperin	*Ericaceae*, *Guttifera*, and *Celastraceae*	Cisplatin-induced AKI	Attenuating inflammatory.	By inhibiting NF-κB and activating nuclear factor E2-related factor-2 signaling pathways (Chao et al., 2016)
Honokiol	*Magnolia officinalis*	LPS-induced AKI	Inhibition of oxidative stress and Inflammation.	By inhibiting TLR2/4/MyD88 signaling pathway (Xia et al., 2019)
Isoacteoside (ISO)	*Monochasma savatieri*	LPS-induced AKI	Attenuating inflammatory.	By inhibiting TLR4 dimerization to activate the MyD88-TAK1- NF-κB/MAPK signaling cascades and TRIF pathway (Gao et al., 2017)
Leonurine (LEO)	*Leonurus cardiaca*	LPS-induced renal injury	Inhibiting inflammatory and oxidative stress.	By down-regulating NF-κB (Xu et al., 2014)
Ligustrazine (LIG)	*Ligusticum wallichii* Franch.	Cisplatin/I/R-induced renal injury	Down-regulating oxidative stress and apoptosis, decreasing neutrophils infiltration.	Liu et al., 2008; Feng et al., 2011
		Pancreatitis-induced AKI	Improving renal function.	By improve microcirculatory disorder (MCD) (Zhang et al., 2006)
Loganetin	*Loganin*	Rhabdomyolysis-induced AKI	Improving renal function.	By inhibiting TLR4 activity and blocking the JNK/p38 pathway (Li et al., 2019)
Luteolin	*Celery*, *Green pepper*, *and Chamomile*	D-galactose-induced AKI	Attenuating inflammatory and oxidative stress.	By suppressing phosphorylation of p38 MAPK (Xu et al., 2015)
		Cisplatin-induced AKI	Alleviating inflammation.	By inhibiting NF-κB (Domitrovic et al., 2013)
			Decreasing apoptosis.	By decreasing p53 (Kang et al., 2011)
Nerolidol	*Essential oils*	LPS-induced AKI	Alleviating inflammation.	By inhibiting TLR4-NF-κB signal pathway (Zhang L. et al., 2017)
Osthole	*Cnidium monnieri* (L.) Cusson *fruit*	LPS-induced AKI	Inhibiting inflammation.	By down-regulating NF-κB pathway (Yu et al., 2017)
		I/R-induced renal injury	Abrogating inflammation.	By suppressing JAK2/STAT3 signaling, NF-κB and activating PI3K/Akt signaling (Luo et al., 2016)
Pachymic acid (PA)	*A lanostane-type triterpenoid from Poria cocos*	Sepsis-induced AKI	Inhibiting inflammatory function and antioxidant effect via.	By activating Nrf2/HO-1 pathway (Cai et al., 2017)
Paeonol	*Paeonia moutan* Sims	Endotoxin-induced AKI	Alleviating inflammation.	By inhibiting TLR4-NF-κB signal pathway (Fan et al., 2016)
*Panax quinquefolius* (PQS)	*Panax quinquefolius*	Cisplatin-induced AKI	Suppressing oxidative stress, inflammation, and apoptosis.	By inhibiting Nox4-iNOS, NF-κB-COX-2, and caspase3/9 (Ma et al., 2017)
Paeoniflorin (PF)	*Radix Paeoniae Rubra*	Pancreatitis-induced AKI	Inhibiting inflammation and cell apoptosis.	By inhibiting NF-κB (Wang et al., 2016)
		ConA-induced renal injury	Attenuating inflammatory response.	By inhibiting CXCR3/CXCL11 (Liu C. et al., 2015)
Panaxadiol Saponin (PDS)	*Ginseng stem and leaves*	LPS-induced AKI	Inhibiting inflammatory and oxidative stress.	By blocking NF-κB pathway (Chen Y. et al., 2015)
Panax notoginseng saponins (PNS)	*Panax notoginseng*	Cisplatin-induced AKI	Reducing renal tissue apoptosis.	By inhibiting the mitochondrial apoptosis (Liu et al., 2014)
			Increasing mitochondrial autophagy.	By enhancing HIF-1α/BNIP3 (Liu X. et al., 2015)
Notoginsenoside R1 (NR1)		I/R-induced renal injury	Blocking apoptosis and inflammatory response.	By suppressing p38 and NF-κB (Liu et al., 2010)
Polydatin (PD)	*Polygonum cuspidatum* Sieb.	I/R, Sepsis-induced AKI	Attenuating inflammatory response	By regulating TLR4/NF-κB and enhancing PI3K/Akt (Liu H. et al., 2015)
Protocatechuic Aldehyde (PA)	*Salvia miltiorrhiza* (*Lamiaceae*)	Cisplatin-induced AKI	Suppressing Nox-mediated oxidative stress and renal inflammation.	By suppressing Nox-mediated oxidative stress targeting RIPK1-mediated necroptosis (Gao L. et al., 2016)
Quercetin (QC)	*Bioflavonoids in the plant kingdom*	I/R-induced AKI	Activating autophagy	By increasing AMPK (Chen et al., 2014)
		HgCl_2_-induced AKI	Limiting apoptosis.	Shin et al., 2015
		Cisplatin-induced AKI	Decreasing cell necrosis and inflammatory.	By inhibiting NF-κB (Francescato et al., 2004)
RA-X II	*Rubia yunnanensis*	LPS-induced AKI	Inhibiting oxidative stress and inflammatory.	By suppressing NF-κB and MAPKs regulated by HO-1/Nrf2 pathway (An and Shang, 2018)
Resveratrol (RSV)	*Grapes and red wine*	LPS-induced AKI	Attenuating inflammatory response.	By NF-B-P65 de-acetylation (Gan et al., 2017), SIRT3-mediated deacetylation of SOD2 (Xu et al., 2016), inhibiting endoplasmic reticulum stress (IRE1)-activated NF-κB pathway (Wang et al., 2017) and via the activation of Nrf2 signaling pathway (Wang et al., 2018)
		Cisplatin-induced AKI	Suppressing inflammation and apoptosis.	By activating SIRT1 through deacetylating p53 (Kim et al., 2011)
		Glycerol-induced AKI	Suppressing inflammatory and lipid peroxidation.	By decreasing NF-κB and HO-1 (de Jesus Soares et al., 2007)
(RSVA405 RSVA314)		As_2_O_3_, I/R -induced AKI	Antagonizing oxidative stress.	Holthoff et al., 2012; Yu et al., 2013
Tanshinone I	*Salvia miltiorrhiza*	AAI-induced renal injury	Inducing apoptosis and autophagy.	By inducing Atg5 (Feng et al., 2013)
Tanshinone IIA		Folic Acid-induced AKI	Inhibiting inflammatory response.	Jiang et al., 2016
Tenuigenin (TNG)	*Polygala tenuifolia*	LPS-induced AKI	Attenuating inflammatory response.	Inhibiting TLR4/NF-κB signaling pathway (Fu et al., 2016)
Tetramethylpyrazine (TMP)	*Ligusticum wallichii* Franch.	Arsenic, Cisplatin-induced AKI	Inhibiting inflammatory and oxidative stress.	By down-regulating HO-1 and ARS2 (Gong et al., 2016)
		Gentamicin-induced AKI	Inhibiting inflammatory and apoptosis.	By enhancing Hax-1 and HO-1 (Sue et al., 2009)
		Sodium arsenite-induced AKI	Suppressing ROS production, mitochondrial dysfunction and inflammatory.	By suppressing programmed cell death (Gong et al., 2015)
		Contrast-induced AKI	Suppressing autophagy and apoptosis.	By suppressing p38 MAPK and targeting FoxO1 (Gong et al., 2013)
		I/R-induced renal injury	Alleviating histopathological damage.	By down-regulating P-selectin (Chen et al., 2003)
Triptolide (PG490-88)	*Tripterygium wilfordii* Hook.F	Cisplatin-induced AKI	Decreasing cell necrosis.	By decreasing phosphorylation of ERK (Kim et al., 2014)
Wogonin	*Scutellaria baicalensis* Georgi	Cisplatin-induced AKI	Attenuating inflammatory response.	By targeting RIPK1-mediated necroptosis (Meng et al., 2018)


### Mechanisms Involved in the Therapeutic Effect of TCMs in AKI

**FIGURE 1:**
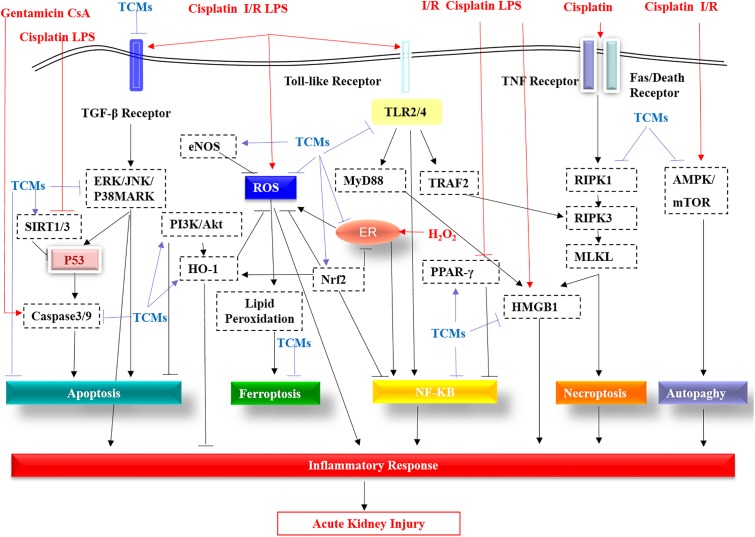
The molecular pathways targeted by the TCMs covered in this review are summarized. TGF-β receptor, Toll-like receptors (TLRs), TNF receptor, and FASL/Death receptors are stimulated by LPS, cisplatin, I/R, etc. These receptors are then activated by the downstream pathway, further triggering ROS production and an inflammatory response, eventually leading to kidney damage. TCMs suppress cisplatin/LPS/I/R-stimulated Toll-like receptors (TLR2/4), or by activating PPAR-γ, further inhibiting the NF-κB pathway and reducing inflammation. Additionally, apart from targeting caspase3/9, TCMs reduce apoptosis by inhibiting the TGF-β receptor, the ERK/JNK/P38MARK pathway and by promoting PI3K/AKT. In addition, TCMs inhibit autophagy by targeting inhibition of AMPK/mTOR. In addition to the traditional apoptosis, autophagy, programmed necrosis and ferroptosis are also caused during AKI. Wogonin and protocatechuic aldehyde can effectively inhibit RIPK1 in the RIPK1/RIPK3/MLKL of necroptosis. TLR2/4-mediated TRAF2 has a stimulant effect on RIPK3. Induction of HMGB2 by necroptosis and TLR2/4-regulated MyD88 aggravates the inflammatory response of acute kidney injury, while TCMs significantly improve this phenomenon via direct or indirect effects. Breviscapine can reduce ferroptosis by increasing glutathione peroxidase levels. In acute kidney injury, the production of ROS, the multiple roles of P53, the protective effects of eNOS, Nrf2, HO-1, and SIRT1/3 all become therapeutic targets of TCMs.

As shown in Figure 1, the TGF-β receptor, Toll-like receptors (TLRs), TNF receptor, and FASL/Death receptors are stimulated by LPS, cisplatin and I/R, etc., then these receptors activate downstream pathways, further triggering ROS production and inflammatory responses, eventually leading to kidney damage. TCMs suppress cisplatin/LPS/I/R-stimulated TLRs including TLR2/4, or by activating PPAR, further inhibiting the NF-κB pathway and reducing inflammation. Moreover, TCMs reduce apoptosis by inhibiting TGF-β, PI3K/AKT and ERK/JNK/P38MARK pathways. In addition, TCMs inhibit autophagy by targeting AMPK/mTOR. In recent years, new cell death mechanisms like programmed necrosis and ferroptosis have also attracted attention. Wogonin and protocatechuic aldehyde can effectively inhibit RIPK1/RIPK3-mediated necroptosis. Breviscapine can reduce ferroptosis by increasing glutathione peroxidase levels. Additionally, TCMs can inhibit H_2_O_2_-induced endoplasmic reticulum (ER) stress and further reduce ROS production. In the AKI model, ROS, HMGB1, P53, Nrf2, HO-1, and SIRT1/3 are regarded as potential therapeutic targets of TCMs.

### Nephrotoxicity of TCMs

It has been recorded that up to 25% of all cases of AKI may be correlated to nephrotoxic medications (Bentley et al., 2010). As shown in Table 3, previous studies indicate that some TCMs, including aristolochic acid, anthraquinones, flavonoids, and glycosides from herbs, non-steroidal anti-inflammatory drugs, aminoglycosides, cytostatic drugs, osmotic agents, radiocontrast, and phosphate salts, may lead to kidney damage and induce AKI (Liangos, 2012; Yang et al., 2018). Several reasons contributing to nephrotoxicity of TCMs include the intrinsic toxicity of herb medicines, incorrect dosing, interactions between herbs and medications, adulteration, incorrect processing and storage, and contamination by heavy metals (Yang et al., 2018).

**Table 3:** Application of TCM monomers which can induce acute kidney injury.

Names	Origins	Functions	Mechanisms
Aristolochia acids (AA)	*Aristolochia species*	Increasing oxidative stress and inflammatory	Increasing Nox2 and reducing NO bioavailability (Sato et al., 2004; Debelle et al., 2008)
Andrographide	*A herbaceous plant in the family Acanthaceae*	Promoting cell necrosis	Unclear (Zhang et al., 2014)
Sciadopitysin	*Taxus celebica*	Inducing acute tubular necrosis and acute interstitial nephritis	(Lin and Ho, 1994)
Triptolide	*Tripterygium wilfordii* Hook.f	Accelerating oxidative stress and inducing apoptosis	Inducing production of ROS (Yang et al., 2012)


It was reported that two patients took sciadopitysin, a kind of flavonoid extracted from *Taxus celebica* to treat diabetes mellitus, and suffered acute tubular necrosis and acute interstitial nephritis (Lin and Ho, 1994). Andrographolide (Chuan Xin Lian) is widely used in China for the treatment of dysentery and respiratory tract infection. It is noteworthy that a systemic analysis, based on clinical cases reported in Chinese literature from January 1978 to August 2013, revealed 26 patients with AKI induced by andrographolide. The major pathologic features in these patients were acute tubular necrosis. The mechanism is still obscure (Zhang et al., 2014). Aristolochia acids (AA) is extracted from *Aristolochia fangchi*, within which aristolochic acid I (AAI) and aristolochic acid II (AAII) are well known. AA has widely been used as an anti-inflammatory agent. However, a series of studies demonstrated that AA could induce early and transient acute tubular necrosis and progressive tubulointerstitial injury, which finally lead to renal fibrosis (Sato et al., 2004; Debelle et al., 2008; Zhou et al., 2010; DeBroe, 2012). AA also caused nephropathy, by inducing DNA adduct formation (Allard et al., 2013). Some drugs related with AA are nephrotoxic due to the intrinsic toxicity of herbs and the misidentification of potentially toxic compounds. The root of asarum (also known as Xi Xin) contains low levels of AA and has widely been used as an analgesic for headache, toothache, and inflammatory diseases. But the whole asarum plant contains high levels of AA (Drew et al., 2002). Triptolide, isolated from *Tripterygium wilfordii* Hook.f (TWHf)-derived diterpenoid, has been commonly used for its immunosuppressive and anti-cancer properties (Carter et al., 2006). However, administration of triptolide may result in severe kidney injury by impairing the antioxidant system, promoting production of reactive oxygen species and inducing apoptosis of tubular epithelial cells, which may limit the application of triptolide in the clinic (Yang et al., 2011, 2012).

### Prevention and Treatment of TCMs-Induced AKI

The first principle of effective therapy is to acknowledge and prevent or minimize nephrotoxicity of TCMs. In this regard, several strategies should be applied: (1) In view of the intrinsic toxicity of some herbs, researchers could modify molecular structure of TCMs to lower the toxic effects without affecting their therapeutic effects. It is essential to reveal the compound/phytochemicals present in the formulations which are correlated with the toxicity in AKI. (2) As for the incorrect identification, processing and storage, standardization of herbal products need to be emphasized. We should also ensure safe manufacturing processes to avoid contamination from heavy metals and other ingredients. (3) We should determine and limit the dosing and duration of drugs usage through adequate preclinical trials and dose conversions between animals and humans. Safe and effective dose ranges for humans as well as appropriate monitoring for adverse effects are also needed. (4) It is worth mentioning that pharmacists and doctors should clearly know the interactions between TCMs and other medications before prescribing these drugs to patients. (5) For patients with special conditions, like chronic kidney disease and liver disease, their medication needs to be carefully considered.

## Conclusion and Perspectives

Collectively, previous studies showed that numerous types of TCMs protect against AKI via different mechanisms of action, including inhibiting inflammation, cell apoptosis, necroptosis, ferroptosis, and restraining oxidative stress etc. These data support the potential application of these TCMs as novel therapeutic agents in treating patients with AKI. Although some TCMs have entered preclinical trials, it is essential to initiate pre-clinical pharmacologic and toxicologic trials and clinical trials to evaluate the efficacy and safety of TCMs usage. Moreover, considering that some TCMs are deleterious to the kidney, they should be attracted more attention when utilized. In addition, it is believed that western medicines always relieve symptoms quickly while TCMs exert therapeutic effects gently and fundamentally. In this regard, the combination of TCMs and western medicines may become a promising treatment strategy for AKI by taking advantages of both and by limiting side effects. The interaction between medicines should also be considered. In conclusion, from a holistic point of view, TCM-based anti-AKI therapy should be emphasized and extensively explored, as this may help to minimize the morbidity and mortality of AKI and prolong the survival of patients.

## Author Contributions

JL, XM, and CH designed the theme and direction of the manuscript. LZ and XL critically revised the manuscript. HL drafted the manuscript.

## Conflict of Interest Statement

The authors declare that the research was conducted in the absence of any commercial or financial relationships that could be construed as a potential conflict of interest.

## Abbreviations

AKI: acute kidney injury
ER stress: endoplasmic reticulum stress
ERK: extracellular signal-regulated kinase
HMGB1: high mobility group box 1
HO-1: heme oxygenase (HO)-1
i.p.: intraperitoneal
I/R: ischemic reperfusion
JNK: C-jun N-terminal kinase
LPS: lipopolysaccharide
MLKL: mixed-lineage kinase like domain
Nrf2: nuclear factor 2 correlation factor
PI3K/AKT: phosphatidylinositol 3-hydroxy kinase/protein kinase
PPAR-γ: peroxisome proliferators-activated receptor-γ
RIPK: receptor interacting serine-threonine protein kinase
ROS: reactive oxygen species
SIRT: silent information regulator
TCMs: traditional Chinese medicines
